# Effects of Interval Jump Rope Exercise Combined with Dark Chocolate Supplementation on Inflammatory Adipokine, Cytokine Concentrations, and Body Composition in Obese Adolescent Boys

**DOI:** 10.3390/nu12103011

**Published:** 2020-09-30

**Authors:** Mozhgan Eskandari, Babak Hooshmand Moghadam, Reza Bagheri, Damoon Ashtary-Larky, Elham Eskandari, Michael Nordvall, Frédéric Dutheil, Alexei Wong

**Affiliations:** 1 Department of Exercise Physiology, University of Birjand, Birjand 9717434765, Iran; Mozhgan.eskandari@birjand.ac.ir; 2 Department of Exercise Physiology, University of Tehran, Tehran 1961733114, Iran; b.hooshmand.m@gmail.com; 3 Department of Exercise Physiology, Ferdowsi University of Mashhad, Mashhad 9177948974, Iran; 4 Department of Exercise Physiology, University of Isfahan, Isfahan 81746-73441, Iran; 5 Nutrition and Metabolic Diseases Research Center, Ahvaz Jundishapur University of Medical Sciences, Ahvaz 61357-15794, Iran; damoon_ashtary@yahoo.com; 6 Qaen school of Nursing and Midwifery, Birjand University of Medical Sciences, Birjand 9717853577, Iran; elhamskandari70@gmail.com; 7 Department of Health and Human Performance, Marymount University, Arlington, VA 22207, USA; michael.nordvall@marymount.edu; 8 Université Clermont Auvergne, CNRS, LaPSCo, Physiological and Psychosocial Stress, CHU Clermont-Ferrand, University Hospital of Clermont-Ferrand, Preventive and Occupational Medicine, Witty Fit, F-63000 Clermont-Ferrand, France; fred_dutheil@yahoo.fr

**Keywords:** fat mass, dark chocolate, obesity, body composition, inflammation

## Abstract

We examined the effects of six weeks of dark chocolate supplementation combined with interval jump rope exercise (JRE) on inflammatory cytokines, adipokines, and body composition in obese adolescent boys. Forty-eight obese adolescent boys (age  = 15.4  ±  1.1 years and body mass index  =  32.2  ±  2.4 kg/m^2^) were randomly assigned into one of four groups: JRE + white chocolate (JW; *n* = 13), JRE + dark chocolate supplementation (JD; *n* = 13), dark chocolate supplementation (DS; *n* = 12), or control (C; *n* = 12). Participants in JW and JD groups performed JRE for three times per week for six weeks. Participants in the DS and JD groups consumed 30 g of dark chocolate containing 83% of cocoa. Body composition, pro-inflammatory cytokines ((hs-CRP, TNF-α, IL-6), adipokines (leptin, resistin, RBP-4, chemerin, MCP-1), and anti-inflammatory adipokines (irisin, adiponectin)) were evaluated prior to and after the intervention trials. All three intervention trials significantly (*p* < 0.05) decreased body mass, waist-hip ratio, fat mass, hs-CRP, TNF-α, IL-6, leptin, resistin, RBP-4, and MCP-1, and increased irisin and adiponectin concentrations. The improvements in these parameters were greater in the JD group, and additionally, chemerin concentrations decreased only in the JD group. JD enhanced adiponectin concentrations and decreased IL-6 concentrations compared to C. Moreover, JD significantly reduced chemerin concentrations, an effect not observed in any of the other interventions. We demonstrated that dark chocolate supplementation potentiated JRE-induced decreases in body mass, WHR, FM, hs-CRP, TNF-α, IL-6, leptin, resistin, RBP-4, and MCP-1, chemerin as well as increases irisin and adiponectin concentrations in obese adolescent boys. Therefore, JRE combined with dark chocolate supplementation could be a beneficial in reducing obesity-induced inflammation in adolescent boys.

## 1. Introduction

It is well established that obesity in youth populations causes alterations in the balance between pro- and anti-inflammatory cytokine and adipokine secretions, resulting in an inflammatory response [1,2]. Indeed, chronic inflammation due to obesity in adolescents and children is a well-known chief risk factor for numerous clinical diseases observed in later adulthood, such as type 2 diabetes mellitus and cardiovascular disease [3].

Numerous physical activity strategies, including jump rope exercise (JRE) [4], have been proposed as a way to combat inflammation and improve health in obese populations [5,6]. For instance, 40 min of daily JRE for six weeks improved insulin sensitivity as well as triglyceride and adiponectin concentrations in obese adolescents [4]. JRE modality requires minimal and inexpensive equipment as well as limited space, which may promote improved exercise adherence in various populations. Additionally, JRE has been proven to reduce inflammatory cytokine and adipokine concentrations in adolescent cohorts, leading to improvements in various body composition and cardiovascular health indices [4,7,8].

Dietary supplements are also receiving substantial interest as a strategy to combat inflammatory-related risk factors in obese populations. A growing body of evidence indicates that cocoa displays beneficial antioxidant and anti-inflammatory properties due to its high flavonoid content including epicatechin, catechin, and proanthocyanidins [9,10]. Indeed, the consumption of food containing cocoa, such as dark chocolate, has been linked to improved health-related markers. For instance, eight weeks of 84% dark chocolate supplementation along with therapeutic lifestyle changes guidelines reduced inflammatory markers (high-sensitivity C-reactive protein (hs-CRP), tumor necrosis factor-alpha (TNF-α), and interleukin-6 (IL-6)) in type 2 diabetic patients [11]. Moreover, eight weeks of dark chocolate supplementation (containing 450 mg polyphenols) decreased hs-CRP concentrations, blood triglyceride, and apo-lipoproteins A-1 and B in diabetic patients [12]. A recent review article indicated that cocoa-rich food intake may lessen inflammation by reducing monocytes and neutrophils activation [13]. Prior research indicates that combining exercise with another anti-inflammatory treatment may offer additive effects of improving circulating concentrations of inflammatory markers [14].

While the anti-inflammatory effects of exercise in obese adolescents are well established [15,16,17,18]; the impact of the combination of JRE and dark chocolate supplementation on inflammatory status in this population has yet to be investigated. Moreover, the effects of chronic dark chocolate consumption on inflammatory markers in obese adolescent cohorts are currently unknown. Therefore, we aimed at investigating the effects of dark chocolate supplementation alone and in combination with JRE on select markers of inflammation (hs-CRP, TNF-α, IL-6, Leptin, Resistin, Monocyte chemoattractant protein-1 (MCP-1), Adiponectin, Chemerin, Irisin, and Retinol-binding protein-4 (RBP-4)). We conducted a randomized, placebo-controlled study to assess the aforementioned effects in obese adolescent boys.

## 2. Materials and Methods

### 2.1. Participants

Forty-eight obese adolescent boys (age  = 15.4  ± 1.1 years and height  =  165.9  ±  4.9 cm) participated in the current study. All participants were obese (body mass index (BMI) equal to or greater than the 95th percentile for age and sex), according to guidelines for youth populations [19]. The inclusion criteria for the study were as follows: ages 13 to 17 years, obesity, weight loss of no more than two kilograms over the last six months, sedentary, participating in less than 1 h of physical activity per week over the last year, and no recognized illnesses, such as cardiovascular disease, diabetes, or similar. All participants were reportedly non-smoking and not consuming alcoholic or caffeine-containing beverages at the time of data collection. Dietary supplementation and drugs affecting muscle mass and adipose tissue metabolism, including amino acids, beta-blockers, beta-agonists, calcium channel blockers, and corticosteroids, were also grounds for exclusion. Information about disease and health status as well as all remaining exclusion criteria was obtained via PAR-Q and appraised by a physician. Participants and their parents provided informed consent before study enrollment. The Institute of Physical Education and Sports Sciences (Tehran, Iran) Human Subject Committee approved the study protocol, which was performed in Mashhad, Iran, and all experimentation was carried out in accordance with the Declaration of Helsinki. The study was registered in the Iranian Registry of Clinical Trials (IRCT20151025024699N5).

### 2.2. Study Design

**Figure 1:**
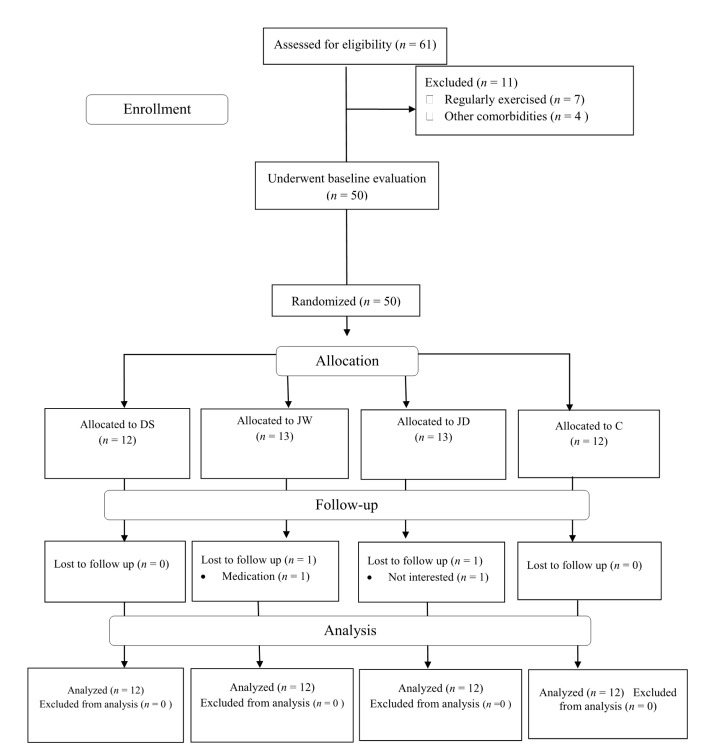
Participants Flow Diagram. Abbreviations: JW, jump rope exercise + white chocolate; JD, jump rope exercise + dark chocolate supplementation; DS, dark chocolate supplementation; C, control.

This study was conducted between January and March of 2019. A schematic of the study design is depicted in Figure 1. Before pre-test measurements, our participants were completely acquainted with all measurements. Participants were randomly assigned into one of four groups as follows: JRE + white chocolate supplementation (JW; *n* = 13), JRE + dark chocolate supplementation (JD; *n* = 13), dark chocolate supplementation (DS; *n* = 12), or control (C; *n* = 12). The randomization assignment was stratified by a digital tool (available at www.randomizer.org). Measurements were collected on two occasions, at baseline and again at 6 weeks post interventions (minimum 48–72 h after the last JRE session). All assessments were performed at the same time of day (within 1 h) and under the same environmental conditions (~20 °C and ~55% humidity). Participants were asked to avoid modifying their normal lifestyle and dietary habits during the study.

### 2.3. Anthropometry and Body Composition

Participants were instructed to fast for 12 h (overnight fast, with at least 8 h of sleep) and abstain from physical activity and alcohol consumption for 48 h prior to anthropometric and body composition data collection. Upon arriving at the laboratory, they were asked to void completely within 30 min prior to data collection. We measured participants’ body mass with a digital scale (Lumbar, Hong Kong, China) to the nearest 0.1 kg. The participant’s height was measured with a stadiometer (Race Industrialization, Shanghai, China) to the nearest 0.1 cm. A multi-frequency bioelectrical impedance analyzer (Jawon Medical X-Contact 356, Seoul, Korea) evaluated body mass index (BMI) and provided estimates of fat mass (FM) and waist-hip ratio (WHR) as previously described [20].

### 2.4. Blood Sampling and Laboratory Analysis

Fasting blood samples (20 mL) were taken from the median cubital vein utilizing standard techniques following 12-h overnight fasting. Blood samples were taken at baseline and 48 h after the final JRE session. Following blood sampling, samples were centrifuged at 3000 rpm for 10 min, and the serum was stored at −80 °C for future analysis of adiponectin (sensitivity: 1.1 ng/mL), leptin (sensitivity: 0.06 ng/mL), resistin (sensitivity: 0.07 ng/mL), Monocyte Chemoattractant Protein-1 (MCP-1; sensitivity: 30.1 pg/mL), Interleukin-6 (IL-6; sensitivity: 2.4 pg/mL), Tumor Necrosis Factor-α (TNF-α; sensitivity: 1.9 pg/mL), hs-CRP (sensitivity: 0.1 ng/mL), chemerin (sensitivity: 7.8 ng/mL), Irisin (sensitivity: 0.7 ng/mL), and Retinol-binding protein-4 (RBP-4; sensitivity: 3.9 ng/mL). Intra and inter-assay coefficient variations for all markers were less than 8% and 10%, respectively (CUSABIO, Houston, TX, USA).

### 2.5. JRE Protocol

JRE was performed five times per week, 40 min/day for 6 weeks. Total exercise duration per session was 40 min and entailed 30 min of JRE as well as warm-up and cool down periods consisting of 5 min of stretching. JRE was performed at a rate of 60 jumps/min for the 1st three weeks and progressed to 90 jumps/min over the remaining 3 weeks. Individual bouts of JRE per exercise session as well progressed over the 6-week study period and were followed by brief rest periods. JRE cadence was established through the utilization of a metronome. All JRE sessions were monitored by personal trainers who verified adherence to the training protocol. The selected JRE protocol is similar to prior research in obese populations [4]. Table 1 represents the JRE protocol.

### 2.6. Dark Chocolate Supplementation

Participants in both JD and DS groups consumed 30 g per day of dark chocolate containing 83% of cocoa (Farmand Gallardo 83 Percent Dark Chocolate) for six weeks. To limit the psychological impact (placebo effect) of not receiving the dark chocolate dietary supplement, participants in the JW group consumed white chocolate of the same shape and packaging (coated with aluminum foil) and devoid of cocoa [21]. Participants received their respective supplementation daily as a midafternoon snack [22]. The ingredients of 30 g of dark and white (placebo) chocolate are shown in Table 2. The dose and timing of the supplementation were similar to a previous investigation reporting the anti-inflammatory effects of dark chocolate [23]. Participants were reminded weekly by phone call and WhatsApp software to consume the dark chocolate supplement in order to maximize study compliance.

### 2.7. Nutrient Intake and Dietary Analysis

Owing to the significant role of nutritional timing surrounding training sessions, dietary intake in the h near (before and after) the JRE sessions were controlled [24]. Our participants consumed a banana that provided 0.30–0.35 g of carbohydrate per kilogram of body mass, as a pre-training snack 1 h before the training session. Moreover, dinner was consumed 1.5–2 h after each exercise session and was standardized to contain 1.7 g/kg of body mass of carbohydrate, 0.3 g/kg of body mass of protein, 0.4 g /kg of body mass of fat [25]. These values were according to recommendations by the Academy of Nutrition and Dietetics, Dietitians of Canada, and the American College of Sports Medicine for macronutrient distribution (55–65% of total calories from carbohydrates, ˂35% of total calories from fats and 10–15% of total calories from protein). Beyond this peri-training nutrient intake standardization, the participants were asked to avoid altering their dietary habits during the study. Our participants were asked to submit 3-day (2 weekdays and 1 weekend) food records at pre-intervention and near the end of the assigned intervention. Each food item was individually entered into the Diet Analysis Plus version 10 program (Cengage, Boston, MA, USA) and total energy consumption as well as the nutrient breakdown of proteins, fats, and carbohydrates, was assessed.

### 2.8. Statistical Analysis

We conducted a priori calculation of sample size utilizing the G*Power analysis software [26]. Our rationale for sample size was according to prior data [4,11] and estimated that 12 participants (48 total) per group would provide 80% power (two-sided α = 0.05) to detect 7% changes in several of our markers (adiponectin, leptin, hs-CRP, and IL-6). However, we attempted to recruit 10% extra participants (5 extra participants) to account for any potential attrition. Data normality was checked using the Shapiro–Wilk test. A one-way analysis of variance (ANOVA) was used for group comparisons at baseline. A two × four ANOVA with repeated measures (time (baseline vs. 6 weeks) × group (JW vs. JD vs.DS vs. C)) with Bonferroni–Holm adjustments was used to determine treatment differences. When a significant group-by-time interaction was presented, one-way ANOVA across change scores was used to detect between-group differences. Statistical significance was set at *p* < 0.05. Cohen’s *d* effect size (ES) was calculated as post-JRE effect mean minus pre-JRE effect mean/pooled pre-JRE effect standard deviation means [27]. An ES of 0.00–0.19 was considered trivial, 0.20–0.49 = small, 0.50–0.79 = moderate, and ≥ 0.80 = large. All analyses were performed using SPSS (version 24.0, IBM; Chicago, IL, USA).

## 3. Results

Between January and March of 2019, we screened 61 obese adolescent boys. Of these only 50 met the criteria for baseline evaluation, and were subsequently randomized to the JW, JD, DS, or C groups. After randomization, one participant in the JW and one participant in the JD group dropped out of the study for health and unwillingness reasons. Compliance with JRE was nearly 92% for participants in both the JW and JD groups. Compliance with dark chocolate supplementation was 100% for participants in the DS group. No adverse events were reported from dark chocolate consumption or JRE. There were no significant differences at baseline between groups for all variables. In addition, there were no significant differences over time or between groups in mean daily energy intake as well as the amount of proteins, fats, and carbohydrates consumed per day (Table 3).

### 3.1. Body Composition

Participant’s anthropometric characteristics, body composition, and inflammatory responses are presented in Table 4. All three intervention groups had significant (*p* < 0.05) decreases in body mass (JW = −1.2 kg (95% confidence interval [CI] = 1.5 to 0.9; ES = 2.2; *p* < 0.001), DS = −0.5 kg (95% CI = −0.7 to −0.3, ES = 1.5, *p* < 0.001), and JD = −2.8 kg (95% CI = −3.1 to −2.4, ES = 5.3, *p* < 0.001)); BMI (JW = −0.4 kg/m^2^ (95% CI = −0.5 to −0.3, ES = 2.4, *p* < 0.001), DS = −0.2 kg/m^2^ (95% CI = −0.3 to −0.1, ES = 1.2, *p* = 0.001), and JD = −1 kg/m^2^ (95% CI = −1.1 to −0.8, ES = 5.5, *p* < 0.001)); WHR [JW = −0.006 m (95% CI = −0.01 to −0.002, ES = 1.1, *p* = 0.005), DS = −0.003 m (95% CI = −0.006 to 0, ES = 0.7, *p* = 0.039) and JD = −0.01 m (95% CI = −0.01 to 0.01, ES = 2.5, *p* < 0.001)); and FM (JW = −0.2 kg (95% CI = −0.4 to −0.1, ES = 0.1, *p* < 0.001), DS = −0.1 kg (95% CI = −0.1 to 0.09, ES = 2.1), *p* < 0.001), and JD = −0.6 kg (95% CI = −0.7 to −0.5, ES = 4, *p* < 0.001)) over time. No alterations in these variables were detected in the C group.

### 3.2. Pro-Inflammatory Cytokines

**Figure 2:**
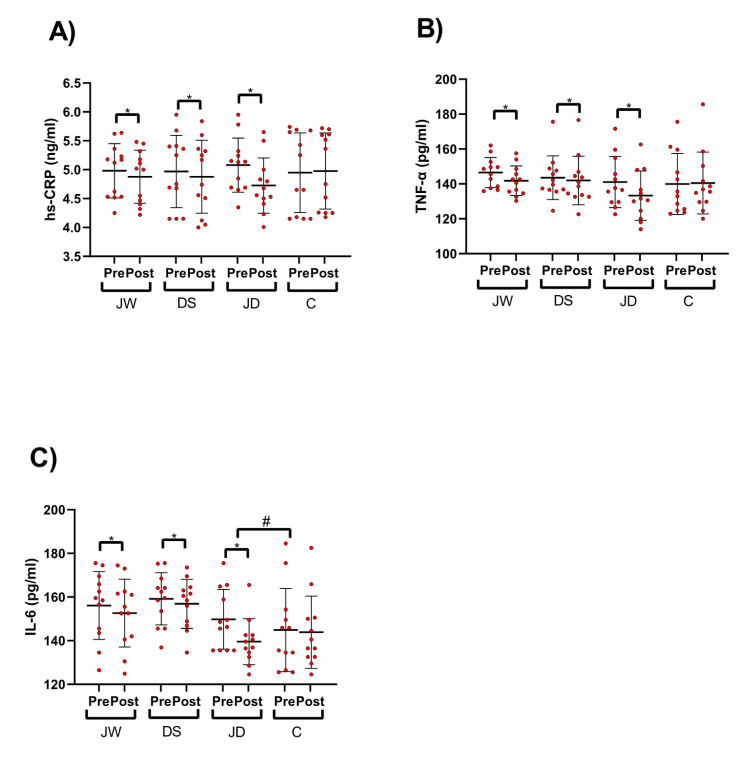
Pro-inflammatory cytokine concentrations. (**A**) high-sensitivity-reactive protein (hs-CRP), (**B**) tumor necrosis factor-α (TNF- α), (**C**) Interleukin-6 (IL-6). * *p* < 0.05 different from pre-training; # between group difference.

Figure 2 depicts pro-inflammatory cytokine concentrations. All 3 intervention groups had significantly (*p* < 0.05) reduced hs-CRP (JW= −0.1 ng/mL (95% CI = −0.1 to −0.06, ES = 1.6, *p* < 0.001), DS = −0.09 ng/mL (95% CI = −0.1 to −0.06, ES = 2.2, *p* < 0.001), and JD = −0.3 ng/mL (95% CI = −0.4 to −0.2, ES = 3.5, *p* < 0.001)); TNF-α (JW = −4.7 pg/mL (95% CI = −6.7 to −2.6, ES = 1.4, *p* < 0.001), DS = −1.5 pg/mL (95% CI = −3.1 to −0.04, ES = 0.6, *p* = 0.045), and JD = −7.7 pg/mL (95% CI = −9.3 to −6.1, ES = 3.1, *p* < 0.001)); and IL-6 (JW = −3.4 pg/mL (95% CI = −4.8 to −2.1, ES = 1.5, *p* < 0.001), DS = −2.3 pg/mL (95% CI = −4.1 to −0.5, ES = 0.8, *p* = 0.016), and JD = −10.2 pg/mL (95% CI = −14.5 to −5.9, ES = 1.6, *p* < 0.001)) over time. No alterations in these variables were detected in the C group. These decreases in the JD group was greater than other groups. There was a significant between-group difference for diminished IL-6 concentrations in JD compared to C group.

### 3.3. Pro-Inflammatory and Anti-Inflammatory Adipokines

**Figure 3:**
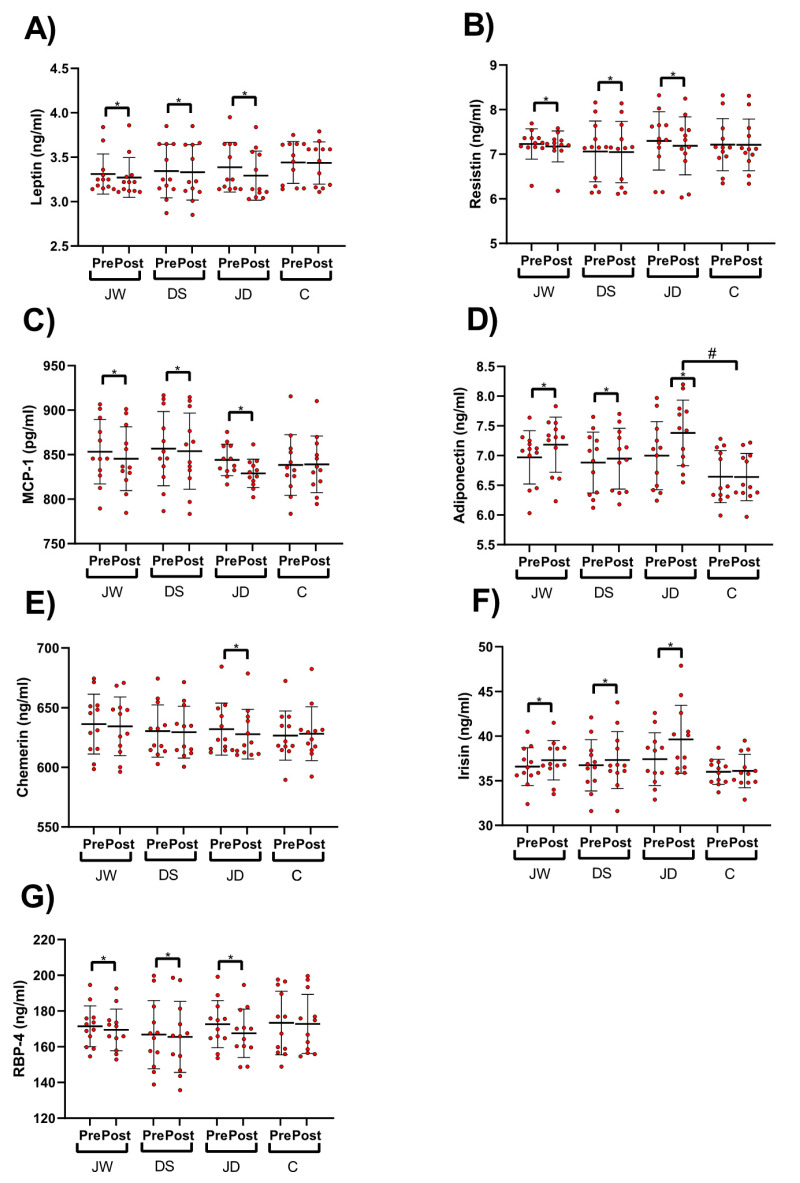
Pro-Inflammatory and anti-inflammatory adipokines concentrations. (**A**) Leptin, (**B**) Resistin. (**C**) Monocyte chemoattractant protein-1 (MCP-1), (**D**) Adiponectin, (**E**) Chemerin, (**F**) Irisin, (**G**) Retinol-binding protein-4 (RBP-4). * *p* < 0.05 different from pre-training; # between group difference.

Adipokines concentrations are shown in Figure 3. All 3 intervention groups demonstrated significantly (*p* < 0.05) reduced leptin (JW = −0.03 ng/mL (95% CI = −0.06 to −0.01, ES = 1, *p* = 0.003), DS = −0.01 ng/mL (95% CI = −0.02 to −0.004, ES = 1, *p* = 0.008), and JD = −0.09 ng/mL (95% CI = −0.1 to −0.07, ES = 4.5, *p* < 0.001)); resistin (JW = −0.05 ng/mL (95% CI = −0.08 to −0.02, ES = 0.1, *p* = 0.003), DS = −0.01 ng/mL (95% CI = −0.02 to −0.002, ES = 1, *p* = 0.021), and JD = −0.1 ng/mL (95% CI, -0.1 to −0.07, ES = 2.2, *p* < 0.001)); MCP-1 (JW = −7.8 pg/mL (95% CI = −9.9 to −5.6), ES = 2.3, *p* < 0.001), DS = −2.8 pg/mL (95% CI = −5.5 to 0.1, ES = 0.6, *p* = 0.041), and JD = −15.1 pg/mL (95% CI = −17.9 to −12.2, ES = 3.3, *p* < 0.001)); and RBP-4 (JW = −1.9 g/mL (95% CI = −2.5 to −1.3, ES = 2.2, *p* < 0.001), DS = −1.2 ng/mL (95% CI = −2 to −0.4, ES = 1, *p* = 0.004), and JD = −5.1 ng/mL (95% CI, -5.9 to −4.3, ES = 4, *p* < 0.001)) over time. No alterations in these variables were detected in the C group. Only Chemerin (−4.2 ng/mL (95% CI = −5.2 to −3.2, ES = 2.7, *p* < 0.001)) concentrations significantly diminished (*p* < 0.05) only in the JD group. 

Anti-inflammatory adipokine concentrations were altered in all 3 intervention groups over time whereby significant (*p* < 0.05) increases in irisin (JW = 0.7 ng/mL (95% CI = 0.3 to 1.1, ES = 1.1, *p* = 0.002), DS = 0.5 ng/mL (95% CI = 0.1 to 1, ES = 0.8, *p* = 0.012), and JD = 2.2 ng/mL (95% CI = 1.2 to 3.2, ES = 1.4, *p* < 0.001)) and adiponectin (JW = 0.2 ng/mL (95% CI= 0.1 to 0.2), ES = 5, *p* < 0.001), DS = 0.06 ng/mL (95% CI = 0.03 to 0.09, ES = 1.5, *p* < 0.001), and JD = 0.3 ng/mL (95% CI = 0.2 to 0.4, ES = 2.9, *p* < 0.001) were observed. No changes were identified in the C group. There was a significant between-group difference for improved adiponectin concentrations in JD compared to C group. 

## 4. Discussion

We aimed at evaluating the effects of JRE following dark chocolate supplementation on pro-inflammatory cytokines (hs-CRP, TNF-α, IL-6), adipokines (leptin, resistin, RBP-4, chemerin, MCP-1), and anti-inflammatory adipokines (irisin, adiponectin) in obese adolescent boys. To the best of our knowledge, our study is the first to assess the influence of combining JRE and dark chocolate supplementation on inflammatory markers in this population. Moreover, this study provides for the novel evaluation of RBP-4, chemerin, and resistin after JRE or dark chocolate supplementation. Briefly, our three main findings were as follows: (1) All intervention groups elicited significant reductions in hs-CRP, IL-6, leptin, resistin, MCP-1, and RBP-4 as well as significant increases in irisin and adiponectin concentrations and these alterations were greater in the JD group. (2) Chemerin concentrations significantly decreased only in the JD group, while no changes were observed in all other interventions and the C group. (3) Although all three interventions significantly decreased body mass, BMI, WHR, and FM, no significant between group changes were observed.

Adiponectin known as a hormone secreted mainly by adipose tissue and targets tissues such as skeletal muscle and liver. Among other functions, it controls metabolism of energy and insulin sensitivity [28,29]. It has been revealed that adiponectin concentrations increased significantly following both high- and moderate-intensity interval training in obese young females [30]. Furthermore, both animal and human studies showed possible effects of cocoa and dark chocolate supplementation on both adiponectin circulating concentrations and gene expression [31,32]. Although there is a shred of evidence on the positive influences of both exercise and dark chocolate supplementation on circulating concentrations of adiponectin, the combination of these two treatments has been insufficiently researched. Our findings underlined that JRE and dark chocolate supplementation either alone or in combination increased adiponectin concentrations, however, the JD induced rise in adiponectin concentrations was greatest compared to C group. A possible mechanism of how dark chocolate supplementation and/or JRE affect adiponectin concentrations may be explained by the observed reduction in adiposity [31] and subsequent catechin-induced promotion of β-oxidation in the liver [33]. In addition, it has been shown that flavonoids in the dark chocolate act on peroxisome proliferator-activated receptors (PPARs) [34] and also serve to increase expression of peroxisome proliferator-activated receptor-gamma (PPAR-γ) which in turn may increase adiponectin expression [34]. It is also understood that adiponectin has an inverse association with BMI and FM [35] which as explained is influenced by physical activity levels [36,37]. Prior research demonstrated that the alterations of adiponectin concentrations were related to decrements of body fat as a result of both high- and moderate-intensity interval training [30]. Therefore, the resultant and heightened changes in adiponectin concentrations in the JD group may partially account for greater decreases in FM. Because adiponectin concentrations also increased in the JW group, it is important to note that traditional aerobic [38] and resistance exercise [39] have not altered adiponectin concentrations in obese adolescents, suggesting that JRE may have a greater influence on this important adipokine.

The main source of pro-inflammatory cytokine secretion is adipose tissue. It has been reported that both JRE and dark chocolate supplementation reduces concentrations of pro-inflammatory cytokines [7,40,41]. For instance, Sung et al. showed that 12 weeks of JRE decreased systemic inflammation by reducing hs-CRP concentrations in prehypertensive adolescent girls [7]. However, six weeks of JRE (40 min/d, 5 d/wk) failed to induce any significant changes in TNF-α, IL-6, and hs-CRP concentrations in obese male adolescents [4]. With respect to the inflammatory responses following dark chocolate or cocoa consumption, the possible effects on pro-inflammatory cytokines remain inconsistent in the literature [10]. It has been previously shown that individuals who received dark chocolate in combination with therapeutic lifestyle changes had lower concentrations of inflammatory markers such as hs-CRP, TNF-α, and IL-6 compared to therapeutic lifestyle changes alone [11]. Conversely, 40 g of daily cocoa powder supplementation decreased the expression of Very-late Antigen-4 (VLA-4), Cluster of differentiation 40 (CD40), and Cluster of differentiation 36 (CD36) in monocytes while circulating pro-inflammatory cytokines, including IL-6 and hs-CRP concentrations, remained unchanged in patients with high risk for cardiovascular disease [42]. Such discrepancies in the literature may be due to differences in the participant characteristics, type, the dose of cocoa and/or dark chocolate, timing and duration of intervention, and inherent methodological variability in quantifying circulating concentrations of cytokines [10,43]. Nevertheless, our results indicated that a combination of JRE and dark chocolate supplementation significantly attenuates pro-inflammatory cytokines, including hs-CRP, TNF-α, and IL-6 concentrations, more than either intervention alone.

Adipose tissue-secreted hormones such as leptin and resistin have significant roles in the initiation and progression of many metabolic disorders such as obesity and diabetes [44]. Previous studies showed a positive association between obesity and leptin and resistin concentrations [45,46,47,48]. Moreover, both leptin and resistin may stimulate the production of pro-inflammatory cytokines such as TNF-α and IL-6 via NF-κB activation [49,50,51]. Therefore, decreasing leptin and resistin concentrations may be considered a therapeutic target to reduce the inflammatory state associated with obesity [44]. To the best of our knowledge, no other study has demonstrated the influences of either dark chocolate supplementation or JRE alone on the pro-inflammatory adipokine concentrations of leptin, resistin, RBP-4, MCP-1, and chemerin. Our findings illustrated that either 30 g of daily dark chocolate supplementation or JRE for 6 weeks in obese adolescent boys decreased leptin, resistin, RBP-4, and MCP-1 concentrations, while chemerin concentrations remained unchanged. However, when dark chocolate supplementation and JRE were combined (JD group), chemerin concentrations decreased. Further investigations are needed to assess the influences of dark chocolate and/or cocoa supplementation on pro-inflammatory adipokines. It has been suggested that cocoa supplementation could enhance advantageous anti-inflammatory properties by mediating the inhibition of the nuclear factor kappa B (NF-кB)-dependent transcription pathway and/or interacting with specific cytokines [10]. The NF-кB pathway appears to mediate low-grade inflammation that is involved in the chronic production of pro-inflammatory cytokines and adipokines seen in obesity [52]. Anti-inflammatory effects after a single dose of 40 g of cocoa supplementation with water or milk in healthy participants mediated by the modulation of NF-kB activation and downstream molecules in this pathway reinforce the link between cocoa supplementation and health outcomes [53]. On the other hand, exercise training has been reported to reduce pro-inflammatory adipokines [54] through reductions in visceral fat mass (via decreased adipokines release) and the production of an anti-inflammatory environment observed with bouts of exercise [55].

Chemerin is an adipokine associated with certain chronic diseases such as diabetes and cardiovascular disease [56]. Prior studies have demonstrated that physical activity may elicit a positive effect of reducing chemerin concentrations [57,58]. Saremi et al. demonstrated that chemerin concentrations decreased following 12 weeks of aerobic exercise [58]. However, our outcomes did not indicate any significant change in chemerin concentration with JRE alone as noted in the JW group. These differences may be due to factors including participant characteristics, type and intensity of exercise, and variations in the exercise intervention. To the best of our knowledge, no other study has demonstrated the effects of dark chocolate and/or cocoa supplementation on chemerin concentrations. Our results further indicated that six weeks of dark chocolate supplementation had no effect on chemerin concentrations in obese adolescent boys (although chemerin trended lower). However, dark chocolate supplementation in combination with JRE (JD group) resulted in significantly decreased chemerin concentrations indicating the potential and beneficial ratio of dark chocolate supplementation to JRE as utilized in this study. Further research is needed to assess the influences of dark chocolate or cocoa supplementation with and/or without exercise interventions on chemerin concentrations to clarify the underlying mechanisms of change.

In this study, we observed reduced body mass in all three-intervention groups, with the greatest reduction seen in JD participants. Our observation is consistent with prior research reporting declines in body mass after JRE in adolescent populations with [4,8], and without [7] obesity. Thus, our findings re-emphasize the notion that JRE is an effective intervention for improving body mass. A meta-analysis of 35 randomized control trial studies found that body mass and BMI were reduced following 30 g or higher of cocoa/dark chocolate supplementation per day ingested over 4–8 weeks [59] which is consistent with our findings in the DS participants. Animal studies utilizing cocoa supplementation have shed light on potential mechanisms of reduced body mass through decreased gene expression involved in liver fatty acids synthesis [60], upregulation of uncoupling proteins [61], and inhibition of insulin receptor kinase activity in adipose tissue [61]. Moreover, human dark chocolate supplementation research has shown a decrease in appetite and an increase in satiety [62,63] potentially resulting from the high-fat content of chocolate, which increases gastrointestinal transit time and may raise the concentrations of satiety controlling hormones [64]. In participants supplementing dark chocolate while undertaking JRE in the present study, there appears a summative body mass-reducing effect (−2.8 kg) which may in part be explained by the heightened FM loss in these participants. Nevertheless, there are certain limitations in drawing such a conclusion since FM, as analyzed by the frequently utilized multi-frequency bioelectrical impedance, assumes that body hydration is normalized, constant, and unaffected by overweightness/obesity and other factors [65]. For example, it is understood that body composition is altered with overweightness/obesity, fluid shifts occur during exercise, and fluid intake influences the redistribution of body fluids [66], all of which may influence estimations of FM. These constraints, when considered in our study, may have underestimated FM in participants as similarly noted in prior research [65,67]. Finally, our JRE protocol was safe, practical, and similar to prior investigation in obese adolescents [4], yet we did not monitor exercise intensity using established methods such as the rating of perceived exertion or the percentage of heart rate or heart rate reserve; however, the American College of Sports Medicine does not indicate such monitoring as part of their current exercise recommendations for children and adolescents [68].

There are some limitations to the present research. We have not utilized the gold standard technique (Dual-energy X-ray absorptiometry (DEXA)) to evaluate body composition. However, previous studies have shown that bioelectrical impedance it is a valid and reliable method [69,70]. The short duration of our intervention is another limitation. Although the total caloric values of the dark and white chocolate supplementations were somewhat similar, macronutrient composition was not evenly matched; which could have, at least to a certain extent, impacted our results. Moreover, the addition of a white chocolate (placebo) supplementation combined with no exercise group would have strengthened the design of our investigation. However, it could be argued that the inclusion of such a group would result in an elevated risk of “non-treatment” as a control group is already present our study. Addressing these four limitations warrants further studies with a larger population and longer duration.

## 5. Conclusions

This study demonstrated that dark chocolate supplementation potentiated JRE-induced decreases in body mass, WHR, FM, hs-CRP, TNF-α, IL-6, leptin, resistin, RBP-4, and MCP-1, as well as increases irisin and adiponectin concentrations. JRE by itself was unable to alter serum concentrations of chemerin, however, when combined with dark chocolate, chemerin concentrations decreased in obese adolescent boys. Based on our results, the combination of JRE with dark chocolate supplementation could be a beneficial strategy to moderate obesity-induced inflammation in adolescent boys. Further longer-term studies are needed to confirm our results.

## Figures and Tables

**Table 1:** Jump rope exercise protocol for the JW and JD groups.

Week	Intensity (Jump/min)	Training Duration		
		Warm-Up (5 min)	Exercise (30 min)	Cool-Down (5 min)
1	60	Stretching	20 sets of 1 min of exercise, followed by 30 s of rest	Stretching
2	60		15 sets of 1.5 min of exercise, followed by 30 s of rest	
3	60		12 sets of 2 min of exercise, followed by 30 s of rest	
4	90		10 sets of 2.5 min of exercise, followed by 30 s of rest	
5	90		9 sets of 3 min of exercise, followed by 30 s of rest	
6	90		7 sets of 4 min of exercise, followed by 30 s of rest	

JW, jump rope exercise + white chocolate supplementation; JD, jump rope exercise + dark chocolate supplementation.

**Table 2:** Energy and nutrient composition of both dark and white chocolate supplements.

Content Per Dose	Dark Chocolate	White Chocolate
Energy (kcal)	184.5	168.8
Total fat (g)	14.6	10.7
Carbohydrate (g)	5.1	14.7
Protein (g)	8.2	3.4
Cacao polyphenol (mg)	2650	0
Epicatechin (mg)	160	0
Caffeine (mg)	130	0
Theobromine (mg)	960	0
Cocoa (%)	83	0

Abbreviations: kcal, kilocalorie; g, gram; mg, milligram.

**Table 3:** Energy and macronutrients at baseline after 6 weeks.

Variable	Group	Baseline	Post-Intervention	P
Energy (kcal/d)	JW	1969.8 ± 45.1	1957 ± 42.3	0.384
	DS	1958.7 ± 71.2	1953.1 ± 69.7	0.540
	JD	1982.5 ± 58.6	1968.8 ± 43	0.428
	C	1959.1 ± 44.3	1954 ± 49.2	0.721
Protein (g/d)	JW	94.2 ± 5.1	94.3 ± 6	0.959
	DS	92.5 ± 5.6	93 ± 5.7	0.702
	JD	94.8 ± 4.9	94.2 ± 5.7	0.749
	C	93.2 ± 4.3	93.1 ± 4.2	0.925
Fat (g/d)	JW	69.1 ± 3.2	68 ± 3.5	0.370
	DS	66.4 ± 3	66.1 ± 4.3	0.782
	JD	68.2 ± 3.2	66.8 ± 2.3	0.173
	C	68.1 ± 3.7	68 ± 3.5	0.944
CHO (g/d)	JW	242.5 ± 9.3	241.9 ± 7.1	0.812
	DS	247.6 ± 10.2	246.4 ± 7.7	0.578
	JD	247.2 ± 10.2	247.5 ± 10.1	0.915
	C	243.1 ± 10.4	242.1 ± 7	0.745

Abbreviations: CHO, carbohydrate; Kcal/d, kilocalorie/day; g/d, gram/day; JW, jump rope exercise + white chocolate supplementation; DS, dark chocolate supplementation; JD; C, control. P, *p*-value between baseline and post-intervention. Values are Mean ± SD.

**Table 4:** Physiological characteristics of participants. Data are mean ± SD.

Variables	Group	Baseline	Post-Intervention	Mean Change
Body mass (kg)	JW	87.7 ± 5.6	86.4 ± 5.7 *	−1.2 ± 0.5
	DS	88 ± 4.4	87.5 ± 4.5 *	−0.5 ± 0.3
	JD	89.7 ± 5.6	86.9 ± 5.6 *	−2.8 ± 0.5
	C	90.2 ± 5.7	90.3 ± 5.7	0.02 ± 0.2
BMI (kg/m^2^)	JW	32 ± 2.2	31.5 ± 2.3 *	−0.4 ± 0.2
	DS	31.3 ± 2.3	31.1 ± 2.2 *	−0.2 ± 0.1
	JD	32.4 ± 2.2	31.4 ± 2.3 *	−1 ± 0.1
	C	33.2 ± 2.9	33.2 ± 2.9	0.02 ± 0.1
WHR (m)	JW	0.99 ± 0.03	0.98 ± 0.03 *	−0.006 ± 0.006
	DS	0.98 ± 0.04	0.98 ± 0.04 *	−0.003 ± 0.004
	JD	0.99 ± 0.03	0.97 ± 0.03 *	−0.01 ± 0.004
	C	1 ± 0.02	1 ± 0.02	0.001 ± 0.007
FM (kg)	JW	32 ± 1.8	31.7 ± 1.9 *	−0.2 ± 0.1
	DS	32.2 ± 2.6	32.1 ± 2.5 *	−0.1 ± 0.06
	JD	32.8 ± 1.8	32.1 ± 1.8 *	−0.6 ± 0.1
	C	33 ± 1.9	33.1 ± 1.9	0.02 ± 0.08

Abbreviations: JW, jump rope + placebo; DS, dark chocolate supplementation, JD, jump rope + dark chocolate supplementation; C, control; WHR, waist hip ratio; FM, Fat Mass. * *p* < 0.05 different from pre-training.
